# National Assessment of the Health Extension Program in Ethiopia: Study Protocol and Key Outputs

**DOI:** 10.4314/ejhs.v33i1.2S

**Published:** 2023-04

**Authors:** Yibeltal Kiflie Alemayehu, Girmay Medhin, Alula M Teklu

**Affiliations:** 1 MERQ Consultancy PLC, Addis Ababa, Ethiopia; 2 Department of Health Policy and Management, Jimma University, Jimma, Ethiopia; 3 Department of Global Community Health and Behavioral Sciences, School of Public Health and Tropical Medicine, Tulane University, New Orleans, USA; 4 Aklilu Lemma Institute of Pathobiology, Addis Ababa University

**Keywords:** Health Extension, Ethiopia, Primary Health Care, Community Health Worker, Protocol

## Abstract

**Background:**

The Health Extension Program (HEP) was introduced in 2003 to extend primary health care services by institutionalizing the former volunteer-based village health services. However, this program is not comprehensively evaluated.

**Materials and Methods:**

The 2019 comprehensive national assessment of HEP involved (1) assessment through quantitative and qualitative primary data, (2) a thorough systematic review of the HEP literature, and (3) a synthesis of evidence from the two sources. The assessment included household survey(n=7122), a survey of health extension workers (HEWs) (n=584)_, and an assessment of health posts (HPs)(n=343) and their supervising health centers (HCs)(n=179) from 62 randomly selected woredas. As part of the comprehensive assessment.

**Output and Results:**

The outputs were (a) full and abridged reports, (b) 40 posters, (c) seven published, three under review scientific papers and (d) seven papers in this special issue. During the one-year period preceding the study, 54.8% of women, 32.1% of men, and 21.9% of female youths had at least a one-time interaction with HEWs. HPs and HEWs were universally available. There were critical gaps in the skills and motivation of HEWs and fulfillment of HP standards: 57.3% of HEWs were certified, average satisfaction score of HEWs was 48.6%, and 5.4% of HPs fulfilled equipment standards.

**Conclusions:**

The findings informed policy and program decisions of the Ministry of Health, including the design of the HEP Optimization Roadmap 2020–2035 and the development Health Sector Transformation Plan II. It is also shared with global community through published papers.

## Introduction

The adoption of the Alma-Ata Declaration at the International Conference on Primary Health Care in 1978 paved the way for government and nongovernment actors to act, protect and promote the health of all people. Nations adopted Primary Health Care (PHC) as a strategy for the then goal of achieving health for all by 2000 and beyond (1, 2). Despite the widespread influence of the Declaration on nations' perceptions of health care and PHC, the implementation of measurable actions has been limited in several countries resulting in low coverage of essential services across several low- and middle-income countries (3). With due consideration of the potential value of the principles of PHC and the prevailing gaps in implementation, leaders of various countries recommitted to the promises of the Alma-Ata Declaration at the 2018 Global Conference on Primary Health Care, which adopted the Declaration of Astana (4).

Ethiopia is one of the early adopters of the concept of PHC and a signatory of the Alma-Ata Declaration(1). During the first two decades after the declaration, PHC in Ethiopia was greatly challenged by the inadequacy and misdistribution of health care facilities and health care workers, which were disproportionately concentrated in urban centers, and the suboptimal health-seeking behavior of communities. The Health Extension Program (HEP) was thus introduced in 2003 to extend health services to the community level and create demand for essential health services. The program introduced an innovative approach that institutionalized the formerly volunteer-based village health services (5).

The HEP was started as a nationwide community-based health program involving the training of two female health extension workers (HEWs) per community, recruited from the communities they were intended to serve, and the construction of one community health post (HP) for every 5,000 people at an accessible location. Initially, the program included 16 packages of essential health care services in the areas of family health, disease prevention and control, hygiene and environmental health, and health education delivered at HPs, through home visits, and in other community settings (6,7). Since its introduction, the program has evolved in several ways, as have the national and global priorities and health systems' approaches. The program was adapted to urban and pastoral settings, packages have expanded in number and contents, and behavior change strategies have shifted from the theory of diffusion of innovation, which anticipates natural spread of desired behaviors from innovators and early adopters to the general public, toward a scale-up approach in which the community health program exerts efforts to reach every household in the community through community structures (8,9).

The program has been widely acknowledged as a major success factor for improvements in health outcomes that Ethiopia achieved during the last two decades (9-11). Major health programs in the country also consider the HEP an essential platform for expanding access to a wide range of their services (10-12). Despite these expectations and contributions, stakeholders have in recent years developed diverging views about the current status of the program and have been characterized by signs of deterioration in the performance of the program, which has led to efforts to conduct a rapid assessment of the program and make adjustments to address the gaps identified (9). Previous studies were, however, limited in their geographic coverage, methodological rigor, domains of program performance assessed, or a combination of these aspects (8,10,15-18). Addressing the performance challenges of the HEP required more comprehensive evidence to inform a longer-term evolution of the program. In response, the Ministry of Health, through financial support from the Bill & Melinda Gates Foundation, commissioned an independent assessment of the HEP. Monitoring, Evaluation, Research and Quality Improvement (MERQ) Consultancy PLC, a private research firm in Ethiopia, designed and implemented the assessment.

This national assessment of the HEP was conducted with the objectives of assessing its status, identifying determinants, determining the adequacy of the resources allocated, exploring its contributions and prospects, and identifying challenges and areas of intervention to inform programmatic and policy decisions in the Ethiopian health sector. It involved an in-depth analysis of the HEP using data collected at all levels of the health system, including communities, HPs, health centers (HCs), health offices, regional health bureaus, HEW training institutions, and the Ministry of Health. Existing literature was also systematically reviewed to triangulate the finding with that of primary data.

## Materials and Methods

**Study context and setting**: With an estimated population of 112.1 million in 2019, Ethiopia is the second most populous country in Africa (13). Despite recent increases in rates of urbanization, most of the population (78.8%) still lives in rural settings, with subsistence farming and animal husbandry as the main sources of livelihood. During the data collection period, the country was administratively divided into nine regional states; namely Tigray, Afar, Amhara, Oromia, Somali, Southern Nation Nationalities and Peoples (SNNP), Benishangul-Gumuz (B-G), Gambella, and Harari, and two city administrations; namely, Dire Dawa and Addis Ababa. *In 2020, the region of Sidama was established, bringing the number of regions to 10*. Each region is divided into zones, then woredas nested within zones, and kebeles nested within Woredas. Woredas and kebeles constitute the lowest budgetary unit of the government, and kebele constitutes the lowest government administrative unit (14). The study covered all the regions and city administrations of the country (**Error! Reference source not found.**).

Health services are delivered through a three-tier health care delivery model. The first level, responsible for primary-level care, comprises PHC units (HC with five satellite HPs) and primary hospitals. At the middle level of the tier system, general hospital provides secondary level care, and at the top levels of the tier system, specialized hospitals provide tertiary-level care.

The HEP, with three versions-agrarian, pastoral, and urban-is the most accessible part of the health care delivery system, particularly in rural settings. In 2018, the program was being delivered by 36,633 HEWs in 17,685 HPs (15). Health institutions at different levels of the administrative structure oversee service provision at different levels of the health service delivery model.

**Scope of the assessment**: The study was a national-level assessment. It included the agrarian HEP in the predominantly agrarian woredas of Tigray, Amhara, Oromia, SNNP, Benishangul-Gumuz, Gambella, and Harari; the pastoral HEP in Afar and Somali; and the predominantly pastoral woredas of Oromia and SNNP. The urban HEP assessment included Addis Ababa, Dire Dawa, regional capital cities, and main towns of study woredas that were included in the rural HEP assessment. In-depth investigations were also done to understand the quality of HEW training institutions, role of HEWs in public health emergency management, attrition of HEWs, and cost-effectiveness of HEP. Study respondents were household members in the community survey (wife, husband, young women aged 15-24 years old), HEWs, staff and students of HEWs training institutions, key informants from all levels of the health system and in terms respondents and personnel documents of HEWs. Health posts were also study units for the audit of readiness and service availability.

**Key areas of the assessment**: Guided by the Primary Health Care Performance Initiative's (PHCPI) framework (16), the study assessed domains related to context, inputs, service delivery, outputs, and outcomes. In particular, we assessed (a) the relevance of the HEP service packages and service delivery modalities, (b) the adequacy of resources, (c) the implementation status of the HEP, (d) the population coverage of essential services, (e) the factors associated with the implementation status of the HEP, and (f) the contribution of the HEP to health outcomes in Ethiopia.

**Study design**: A mixed-methods study was employed (17). We synthesized evidence from quantitative and qualitative studies using primary data collected at different levels of the health system and a systematic review of previous studies to comprehensively understand the program's status, factors associated with its status, and its contributions to the gained health outcomes. Primary data collection and systematic review of existing literature were conducted concurrently, and their findings were interpreted together. The quantitative part of the assessment involved a cross-sectional survey of households, HPs, HEWs, and HCs, all nested in a sample of woredas. The household survey involved interviews with women, men, and female youths of the study households. The qualitative study included Key Informant Interviews (KII) with health workers and managers, in-depth interviews with HEWs, and Focus Group Discussions (FGDs) with community members. The systematic review focused on the effectiveness of the HEP or any of its components in improving health outcomes. In addition to conducting the national assessment of agrarian and pastoral areas, we further conducted in-depth studies on five selected topics: (a) urban HEP assessment using cross-sectional quantitative survey and in-depth interview of various actors, (b) HEW training institution assessment using cross-sectional quantitative survey, observation check list and qualitative interviews with the trainee, teachers and managers, (c) attrition and determinants of attrition among HEWs using review of personnel documents pertinent of HEWs, (d) the role of HEP in public health emergency management using cross-sectional quantitative survey and in-depth interviews, and (e) the cost-effectiveness of services provided through the HEP employing cross-sectional quantitative survey method.

A review of the HEP literature was conducted in parallel with the collection and analysis of primary data. The review included national studies like service provision assessment (SPA), service availability and readiness assessment (SARA), emergency obstetric and newborn care assessment (EmONC), the EDHS, other published papers, and gray literature. Different review databases were searched for the presence of a review. Databases included: the Joanna Briggs Institute Database of Systematic Reviews and Implementation Reports, the Cochrane Database of Systematic Reviews, the Campbell Collaboration Library, the National Health Centre Reviews and Dissemination Databases, and Health Technology Assessment, and Evidence for Policy and Practice Information. Search terms used were health extension program, Health Extension Worker, Health Extension Worker motivation, health extension program actors, health extension program implementation, health extension program implementation challenges, health extension program implementation bottlenecks, community health, and community health programs.

**Populations and sampling**: Quantitative data for the main study were collected from members of randomly selected households (i.e. women, men, and female youths whose age is 15-24 years), HEWs, HPs, HCs, and woreda health offices (WoHOs). A randomly selected one young female was interviewed in the study household in a rare case situation of having more than one eligible young female. Qualitative data were collected from community members and experts at different levels of the health system. Sample sizes for quantitative data collection were calculated to determine the coverage of key health services at the population level and the availability of essential inputs at the HP level. A three-stage stratified sampling strategy was employed. In the first stage, we randomly selected 64 woredas (62 included excluding two because of security reasons) from a sampling frame stratified by regions and predominant means of livelihood. In the second and third stages, we selected kebeles and households, respectively. HPs found in each selected kebele and HCs that supervise these HPs were automatically included in the study. Taking cost of travel into account, it was decided to recruit 34 households in each kebele which was then enough to include only three kebeles from each of the 62 woredas. Hence, the household survey was conducted in half of the study kebeles. In kebeles where the household survey was conducted, four randomly selected Women's Development Army (WDA) leaders were included as an independent sample. Accordingly, we included 343 HPs, 584 HEWs, 179 HCs, 6,504 households in the general population, and 618 households of WDA leaders. Qualitative data were collected through 172 KIIs and 109 FGDs. Additional respondents were involved in the in-depth studies that focused on specific topics (Table 1).

**Table 1:** Study participants and sample size

Study Component	Study Population – Quantitative	Study Population – Qualitative
**Main study – rural HEP** assessment	6,430 women, 4,416 men, and 900 female youths from 6,504 households in the general population 613 women, 389 men, and 120 female youths from 618 households of WDA leaders 343 HPs, 584 HEWs, 179 catchment HCs, and 62 WoHOs	109 FGDs and 172 KIIs: men and community leaders = 22, female community members = 29, WDAs = 52, HEWs = 38, kebele leaders = 13, HC heads and supervisors = 49, WoHO = 40, regional level experts = 25, Ministry of Health (MoH) = 5, partners = 6, policy advisors = 2
**Urban HEP assessment**	Addis Ababa: 98 HCs, 404 Urban HEWs, and 1,287 female community members Dire Dawa: 7 HCs, 87 urban HEWs, and 625 female community members Woreda towns: 34 HCs and 113 urban HEWs	57 FGDs and 62 KIIs: WDA leaders = 13, service user community members = 22, nonuser community members = 6, urban HEWs = 16, HC staff = 32, WoHO staff = 4, sub-city health office staff = 11, city health bureau staff = 15
**Training institution assessment**	21 colleges 192 instructors 1,245 trainees	43 KIIs: 3 deans, 7 department heads, 7 instructors, 9 trainees, 7 preceptors, 6 Certificate of competency (COC) assessors, and 4 focal persons Document reviews: curriculum, teaching materials, standards, and policies from each college
**Attrition and determinants of attrition among HEWs**	Personnel records of 3,476 HEWs who had ever been deployed	In-depth interviews: 8 HEWs who left the system
**The role of HEP in public health emergency management**	96 HPs 142 HEWs 52 HCs 54 WoHOs	
**Cost-effectiveness of services provided through the HEP**	Cost and effectiveness data were constructed using secondary data from different sources.	

**Development of data collection tools**: Data collection tools were developed to guide the collection of quantitative and qualitative data. Quantitative data collection tools for the main study included: (a) household questionnaire with separate modules for household, women, men, and female youths; (b) HP assessment tool, (c) HEW survey questionnaire, (d) HC assessment tool, and (e) WoHO HEP assessment tool. Topic guides for qualitative data collection using KII and FGD were adapted for different categories of respondents.

Data collection tools were prepared through a process that involved four key steps: identification of sub-constructs related to domains of the PHCPI framework through literature review; determining data need at federal, regional, woreda, HC, HP, and community levels for each of the identified sub-construct; adopting standard questions, whenever available, from different sources for each of the data need; and formulating new questions for data needs with no preexisting source of standard questions such as Demographic Health Survey and Service Availability Readiness Assessment.

Questions were then arranged into data collection tools and topic guides based on their respective data sources. All survey tools were translated into six local languages (Amharic, Afan Oromo, Tigrigna, Somali, Nuer, and Afar) and then translated back into English to ensure accuracy. The translated tools were pretested in communities outside of the sample woredas prior to data collection. All quantitative data collection tools were programmed for electronic data collection using Open Data Kit (ODK).

**Data collection**: Primary data were collected from March to May 2019 in two phases. The first phase focused on data required for assessment of the HEP in agrarian and pastoral settings, whereas the second phase emphasized the data needs of in-depth studies on specific topics (i.e. urban HEP assessment, HEW training institution assessment, attrition and determinants of attrition among HEWs, the role of HEP in public health emergency management and the cost-effectiveness of services provided through the HEP). The reasons for having phased approach were (a) to insure quality of data collection by limiting filed activities, and (b) to incorporate our learning from the first phase to some issues in the 2^nd^ phase. Data were collected through household surveys, HP assessments, surveys of HEWs, health institution assessments, assessments of HEWs training institutions, assessments of the documentation pertaining to HEW personnel, and KIIs and FGDs with various stakeholders. Household data were collected through face-to-face interviews with eligible respondents. Household characteristics were assessed for all sample households, mostly through interviews with women. The remaining modules were completed based on the availability of an eligible respondent in the household. In addition to the household survey in the general population, data were also collected from four households of WDA leaders in each of the kebeles where household survey was conducted. Most of the information required for cost-befit analysis was included in the household questionnaire. The remaining data were collected during the 2^nd^ phase of the assessment.

HP assessments were conducted by interviewing heads of HPs, making observations in the HPs, and reviewing HP records. In each of the HPs included in the assessment, all on-duty HEWs were interviewed, with a focus on factors that are potentially related to the performance of the HEP. Health institution assessments at HC and WoHO levels were completed by reviewing documents and interviewing health workers responsible for overseeing HEP in their respective catchment areas. The assessments were focused only on the HEP-related functions of the institutions.

Data related to attrition of HEWs were extracted from personnel fields of HEWs with the help of woreda level personnel offices in most of the cases and zonal or regional level personnel office in rare cases. Qualitative data from HEWs teaching institutions were collected by interviewing all available teachers and randomly selected HEW trainees.

Qualitative data were collected through FGDs and KIIs. At the community level, FGDs were facilitated with WDA leaders, community leaders, and men and women from the general population. FGDs were also held among health promotion, disease prevention, and maternal and child health program experts who had been using the HEP as a platform to implement their programs. KIIs were held with kebele administrators, HEWs, and experts at all levels of the health system. KIIs were also held with heads of HEWs training institutions, teachers in these institutions and the trainees. The audio from each FGD and KII was recorded to ensure its accurate and complete transcription.

**Data quality assurance procedures**: Data quality assurance procedures included actions taken before the start of field data collection, during field data collection, and after completing field data collection. Actions taken before the start of data collection include integration of data validation rules in ODK data collection templates, recruitment of experienced data collectors, and provision of 10-day-long intensive training for data collectors and supervisors. During data collection, standard operating procedures were used as field guides for data collectors and supervisors. Supervisors checked the quality of data on daily basis; a central data quality assurance team regularly checked the consistency and plausibility of data submitted to a central server and provided feedback to the field team. Spot checks were also conducted at a sample data collection sites. After completion of the data collection, a final quality check was conducted upon handover of the data collection equipment, during which data on the central server were compared with individual tablet computers, and explanations regarding missing or unexpected values were obtained from the field team.

In addition to the general quality assurance procedures applied to both quantitative and qualitative data collection activities; we checked the quality of qualitative data through random checks on the quality of the transcripts and translations of audio records from KIIs and FGDs.

### Data analyses

**Quantitative data analyses**: For the purpose of producing comprehensive report from all components of the assessment, we used descriptive statistics with regional and livelihood disaggregation. This helped us to determine the implementation status of the HEP and to generate estimates of various indicators. Because of the disproportionate sample size allocation in different regions and the multistage sampling strategy used to recruit study participants, aggregate-level estimates involved appropriate weighting. The weights were calculated considering multistage sampling and in such a way that the number of eligible target populations represented by each study unit was considered. The population size of each region, the number of woredas in each region, the number of kebeles in each woreda, and the number of households in each of the selected kebeles were documented during the household survey. This information was used to calculate the weights to be used in the analysis of household, HP, and HEW data while generating national and sub-national estimates. The weights for kebeles, HPs, and households were calculated as the inverse of their respective probabilities of selection. In different scientific papers produced and being drafted based on the data from the HEP assessment, various regression models used the generated weights and survey commands as implemented in STATA version 16. In these papers including the sevenpapers reported in the current special issue, different indicators of HEP performance were considered as outcome variables, and factors associated with the outcomes were investigated.

**Qualitative data analyses**: Transcripts of audio records from KIIs and FGDs were entered into NVivo version 12 qualitative data management software and analyzed thematically. A codebook was developed by considering the objectives of the study, the conceptual framework of the study, the contents of the data collection guides, and the contents of sample transcripts. All transcripts were coded using the developed codebook. Once the coding was complete, code reports were produced for each code, cleaned, and prepared for synthesis. For several papers that are planned to be drafted and published using qualitative data alone or using mixed methods, relevant code reports were synthesized and summarized by qualified coauthors of each paper. Illustrative quotes were identified, as necessary. For papers that will be designed to follow a mixed-methods approach, synthesized contents were presented in narrations, allowing triangulation and complementing the findings derived from quantitative data.

**Systematic review**: Systematic review was conducted to assess the contribution of the HEP to health outcomes in Ethiopia. Relevant articles were searched on databases of peer-reviewed journals and the gray literature using different strategies and engines—PubMed, Cochrane Library, Medline, Web of Science, and Google Scholar. Reports and gray literature, including program documents, student thesis reports, policies, and other strategic documents were included in the initial list of contents for review. After removing duplicates, 1,521 articles were identified, of which 45 were included for meta-analysis and an additional 22 were included for qualitative synthesis.

**Synthesis and recommendation formulation workshops**: The national assessment involved a process through which the external team of experts had an opportunity to engage program experts and stakeholders with lived experiences in managing and supporting the program. Synthesis and recommendation formulation workshops were organized to obtain experiential inputs of stakeholders in the interpretation of assessment findings and the formulation of recommendations. Three major workshops were organized. In each workshop, key findings of the assessment were presented to participants, who then discussed implications and possible recommendations. The assessment team documented and used stakeholders' perspectives in synthesizing findings and formulating recommendations.

**Key outputs from the assessment**: The assessment provided a comprehensive appraisal of the HEP in terms of relevance of contents and strategies, availability and adequacy of inputs, implementation status and determinants of implementation, and contributions of the HEP to the health care delivery at a grassroots level so far. It assessed the agrarian, pastoral, and urban versions of the program from the perspectives of communities, HEWs, supervisors of HEWs, and stakeholders at all levels of the health care system. Data were collected from 7,122 households, 343 HPs, 584 HEWS, and 179 HCs from 62 woredas. Tables 1 and 2 present the number of respondents who participated in the assessment through various methods and their regional distribution.

**Table 2:** Number of study participants for the national assessment of the HEP by livelihood and regions

Method	Units/categories of respondents	National	Livelihood						Region					

			Agrarian	Pastoral	Tigray	Afar	Amhara	Oromia	Somali	BG	SNNPR	Gambella	Harari	Federal
Assessments at institutional level	Woredas	62	42	20	6	4	10	13	8	4	10	4	3	
	HPs	343	235	108	32	18	60	74	43	24	59	17	16	
	HCs	179	139	40	27	7	39	46	10	6	35	5	4	
	HEWs	584	414	170	63	19	95	123	75	37	96	42	34	

Households surveyed in the general population	Households	6,50	4,454	2,05	614	41	1,06	1,32	821	40	1,02	422	416	
		4		0		2	6	3		7	3			
	Women	6,43	4,421	2,00	607	39	1,06	1,31	798	40	1,00	417	415	
		0		9		9	0	9		6	9			
	Men	4,41	3,157	1,25	407	27	603	1,13	376	34	759	157	360	
		6		9		5		9		0				
	Youths	900	658	242	117	61	169	162	97	50	150	36	58	

Households surveyed among WDA/SMC households	Households	618	400	218	71	37	71	152	93	46	112	0	36	
	Women	613	400	213	71	35	71	152	90	46	112	0	36	
	Men	389	250	139	25	24	33	121	63	36	60	0	27	
	Youths	120	88	32	16	7	16	30	13	16	17	0	5	

Number of KIIs and FGDs	WDA leaders	52			6	6	9	9	2	6	9	1	4	
	Female community members	29			4	3	4	5	2	3	5	1	2	
	Men and community leaders	22			4	2	4	2	1	3	3	2	1	
	Kebele administrators	13			0	2	2	3	1	2	2	1	0	
	HEWs	38			6	4	6	7	2	4	6	1	2	
	HC heads	25			3	3	4	3	0	3	6	1	2	
	HEP supervisors	24			2	3	6	2	2	2	2	2	3	
	WoHO heads	10			1	1	1	1	2	1	2	0	1	
	WoHO HEP coordinators	13			1		1	2	0	0	4	3	2	
	WoHO process owners	17			2	1	3	3	1	3	0	1	3	
	Regional Health Bureau (RHB) heads	8			1	1	1	1	0	1	1	1	1	
	RHB HEP coordinators	9			1	1	1	1	1	1	1	1	1	
	RHB program officers	8			1	1	1	1	0	1	1	1	1	
	Federal experts and advisors	13												13
	Total	281			32	28	43	40	14	30	42	16	23	13

*Includes only the assessment in rural areas for the main component of the national assessment; does not include specific studies and urban HEP assessment

The outputs are summarized in (a) full and abridged reports, (b) 40 posters, and (c) pre-reviewed scientific publications. To date, seven papers have been published (23-29), and three papers on the areas of WASH, nutrition and job satisfaction of HEWs are under 2^nd^ round review. Six papers focusing on various aspects of HEWs and HP, and one paper deARscribing the protocol of the HEP assessment are the subject of the current special issue.

## Figures and Tables

**Figure 1:**
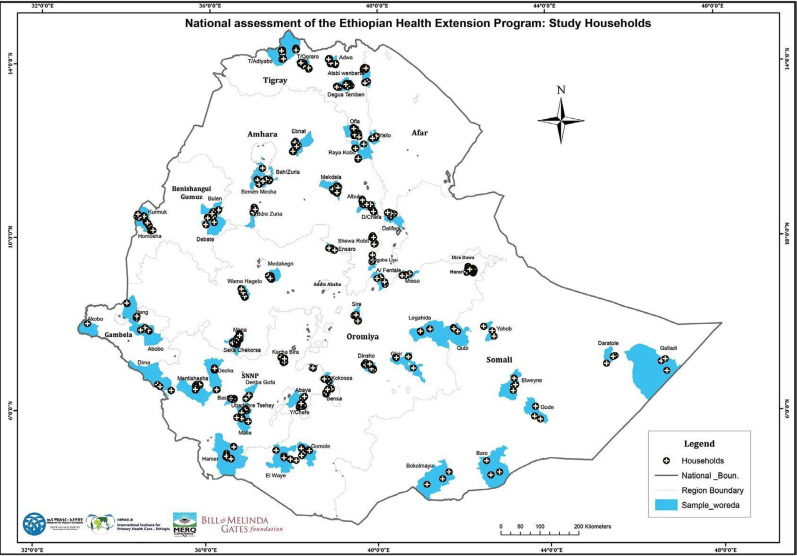
Study sites of the HEP assessment

**Figure 2:**
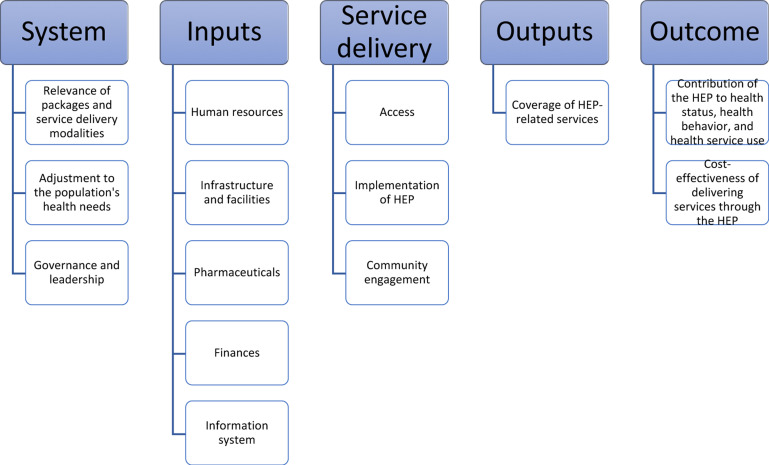
Framework for assessment of the HEP (adapted from the PHCPI framework) (18)
